# Toxicological and Pharmacological Activities of *Leptohyptis macrostachys* (Benth.) Harley and J.F.B.Pastore (Lamiaceae) on Intestinal Smooth Muscle

**DOI:** 10.3389/fphar.2020.01042

**Published:** 2020-07-10

**Authors:** Iara Leão Luna de Souza, Maria Allynne de Vasconcelos, Anne Dayse Soares da Silva, Polyana Cristina Barros Silva, Carlos Arthur Gouveia Veloso, Diego Igor Alves Fernandes de Araújo, Vicente Carlos de Oliveira Costa, Hilzeth Luna Freire Pessôa, Josean Fechine Tavares, Marcelo Sobral da Silva, Bagnólia Araújo da Silva, Fabiana de Andrade Cavalcante

**Affiliations:** ^1^ Departamento de Ciências Biológicas e Saúde, Universidade Estadual de Roraima, Boa Vista, Brazil; ^2^ Instituto de Ciências Biológicas e da Saúde, Universidade Federal de Alagoas, Maceió, Brazil; ^3^ Programa de Pós-graduação em Química e Biotecnologia, Instituto de Química, Universidade Federal de Alagoas, Maceió, Brazil; ^4^ Instituto de Pesquisa em Fármacos e Medicamentos, Universidade Federal da Paraíba, João Pessoa, Brazil; ^5^ Departamento de Biologia Molecular, Centro de Ciências Exatas e da Natureza, Universidade Federal da Paraíba, João Pessoa, Brazil; ^6^ Departamento de Ciências Farmacêuticas, Centro de Ciências da Saúde, Universidade Federal da Paraíba, João Pessoa, Brazil; ^7^ Departamento de Fisiologia e Patologia, Centro de Ciências da Saúde, Universidade Federal da Paraíba, João Pessoa, Brazil

**Keywords:** *Leptohyptis macrostachys*, Lamiaceae, spasmolytic activity, antidiarrheal activity, potassium channel

## Abstract

*Leptohyptis macrostachys*, previously known as *Hyptis macrostachys* Benth., is used in folk medicine to relieve the symptoms of asthma, cough, and bronchitis. Recently, we showed that the ethanol extract obtained from *Leptohyptis macrostachys* has selective spasmolytic activity on guinea pig ileum. Therefore, the aim of this study was to characterize the spasmolytic mechanism of this extract, investigated whether it presents toxicological and antidiarrheal activities. Therefore, the crude ethanolic extract of *Leptohyptis macrostachys* was analyzed by high-performance liquid chromatographic-diode array detection (HPLC–DAD). The spasmolytic effect was evaluated on guinea pig ileum, toxicological activity using rats and antidiarrheal activity using male and female mice. In HPLC-DAD analysis, Rosmarinic acid (5.44%) was the most abundant phenolic compound, being considered as a chemical marker. The spasmolytic potency of the extract on histamine-induced contraction was reduced in the presence of 1 mM TEA^+^, a selective big–conductance K^+^ channels blocker (BK_Ca_). The extract produces a dose–dependent antidiarrheal activity, inhibiting equipotently defecation frequency and liquid stool formation. In addition, the extract has inhibited in a dose–dependent manner both castor oil–induced intestinal transit and intestinal fluid content. Thus, the spasmolytic activity of the extract involves positive modulation of BK_Ca_ and its antidiarrheal activity is related to inhibition of intestinal motility and secretion.

## Introduction

Lamiaceae, also called as the mint family, has a worldwide distribution and includes around 295 genus and 7,780 species (Stevens, 2001). It has species with high economic importance used as cosmetics, condiments, medicines, among others (Sousa and Couri, 2013). *Hyptis*, a highlighter genus of this family, is composed by 400 species with broad distribution in America, Oceania, and Africa (Raja, 2012). This genus has different species that have therapeutic potential such as antifungal (Rocha et al., 2019), antinociceptive, anti-inflammatory (Anjos et al., 2017; Figueiredo et al., 2019), antiedematogenic (Barbosa et al., 2017), antibacterial (Andrade et al., 2017), among others. In addition, several *Hyptis* species have shown spasmolytic and antidiarrheal effects, such as *H. fruticosa*, *H. pectinata*, *H. martiusii* (Agra et al., 2008), *H. suavelons* (Agra et al., 2008; Attah et al., 2012; Shaikat et al., 2012), *H. capitata* (Almtorp et al., 1991), and *H. macrostachys* (Souza et al., 2013; Costa et al., 2014).

*Leptohyptis macrostachys* (Benth.) Harley and J.F.B.Pastore, previously known as *Hyptis macrostachys* Benth., it is a species popularly known in Brazil as “alfavaca-brava” and “hortelã–do–mato,” is used in folk medicine to relieve the symptoms of asthma, cough, and bronchitis (Agra et al., 2008). Recently, we developed a pharmacological screening with the crude ethanolic extract obtained from the aerial parts of *L. macrostachys* (LM-EtOH_AP_) in different smooth muscles (rat aorta, rat uterus, and guinea pig trachea and ileum) and this preliminary study showed a selective spasmolytic effect of the extract on guinea pig ileum (Souza et al., 2013).

Currently, diarrhea treatment involves the use of spasmolytic, anti-motility, anti–secretory, anti–fungi and anti-bacteria agents, and/or oral rehydration therapy. Despite this, the therapy varies in potency, availability of systemic action, availability of the central nervous system, in addition to addictive potential and the potential side effects include constipation, cramps, nausea, and colon ischemia (Schiller, 2017). This disease can be related to viral, bacterial, and fungi infection, food poisoning, among other conditions. Since uncontrolled diarrhea results in severe dehydration and death, the search for potent drugs with antidiarrheal properties is growing, in order to reduce the pattern of adverse effects (Otimenyin and Uzochukwu, 2010).

Therefore, based on the fact that pharmacologic diarrhea therapy can include spasmolytic agents, we decided to better characterize the spasmolytic action mechanism of LM-EtOH_AP_ on guinea pig ileum and investigated whether this extract presents toxicological and antidiarrheal activities.

## Material and Methods

### Plant Material

*L. macrostachys* was collected in Pico do Jabre, Maturéia municipality, Paraíba, Brazil, in March 2009 and identified by Maria de Fátima Agra (PhD) of the Programa de Pós–graduação em Produtos Naturais e Sintéticos Bioativos (PPgPNSB) of Centro de Ciências da Saúde (CCS) of Universidade Federal da Paraíba (UFPB). The voucher specimen is deposited in the Herbarium Prof. Lauro Pires Xavier/UFPB, under the identification code “Agra 6947.”

### Extraction

Plant material was dried in a stove with circulating air (40°C) and chopped by a mechanical mill. The fine powdered from aerial parts (3.0 kg) was macerated with 95% ethanol (5 L) during 72 h. The extraction solution was concentrated under vacuum in a rotaevaporator to give 200 g of ethanol extract of the aerial parts of *L. macrostachys*.

### High-Performance Liquid Chromatography Analysis

Analytical separation was performed using an HPLC‐DAD system consisting of a Prominence Shimadzu LC-20AT quaternary pump, a degasser DGU 20 A-Sr, an auto-injector SIL - 20 A, an oven CTO - 20 A, a photodiode array detector SPD‐M20 A with a CBM-20 A interface, and a Kromasil^®^ 100 column – C-18 (250 × 4.6 mm - 5 μm) protected by a pre-column Security Guard Gemini^®^ C-18. HPLC data acquisition was performed by LC Solution software. The optimized analytical separations of rosmarinic acid were carried out using a mobile phase that consisted of 0.1% formic acid in water (solvent A) and methanol (solvent B) with the following method: 1–12 min: 0 to 40% of B; 12–15 min: 40 to 50% of B; 15–22 min: 50% of B; 22–24 min: 50 to 40% of B; 24–30 min: 40% of B. A flow rate of 1.0 ml/min at 30°C and an injection volume of 20 μl were employed. The UV spectra were recorded at 329 nm. The rosmarinic acid standard (>99%) was purchased by Sigma-Aldrich^®^. The samples were filtered through a 0.45-μm nylon membrane (Tedia Brasil^®^). Seven solutions of different concentrations of rosmarinic acid (10 to 70 μg/ml) were injected in triplicate, and the regression equation and the linearity factor were determined. The LOD was calculated based on a signal-to-noise ratio (S/N) of three, while the LOQ was determined at an S/N of ten. The noise level was measured during the analysis of one of the samples and involved a portion of the chromatogram that was separated from the region containing the rosmarinic acid peak.

### Animals

On experimental protocols were used guinea pigs (*Cavia porcellus*) weighting 350–500 g of both sexes, male Wistar rats (*Rattus norvegicus*) weighting 200–300 g, and Swiss mice (*Mus muscullus*) weighing 25–35 g of both sexes. The mice and male rats were obtained from the Central Bioterium of Universidade Federal de Alagoas (UFAL) and guinea pigs were obtained from the Bioterium “Professor Thomas George” of UFPB. Previously, the animals were maintained in a 12-h light-dark cycle under controlled temperature (21 ± 1°C) and with free access to food and water. The experimental procedures were approved by the Ethics Committee in Research (CEP) of UFAL, certificate no. 006775/2011-09 and the Ethics Committee on Animal Use (CEPA/UFPB), certificate no. 0506/05.

### Chemicals

Magnesium sulphate (MgSO_4_), potassium chloride (KCl), calcium chloride (CaCl_2_), and sodium chloride (NaCl) were purchased from Vetec Química Fina Ltda. (Brazil). Glucose (C_6_H_12_O_6_) and sodium bicarbonate (NaHCO_3_) were purchased from Dinâmica (Brazil). Sodium dihydrogen phosphate (NaH_2_PO_4_) was purchased from Nuclear (Brazil). Histamine, atropine, Cremophor^®^, Triton-X 100^®^, apamin, cesium chloride (CsCl), tetraethylammonium chloride (TEA^+^), 4–aminopyridine (4–AP), and glibenclamide were obtained from Sigma–Aldrich (Brazil). Carboxymethylcellulose and castor oil were obtained from Fórmula (Brazil). Loperamide was obtained from Janssen Cilag Farmacêutica Ltda. (Brazil) and the activated charcoal was obtained from Proquímios (Brazil).

All substances were diluted in distilled water and the extract was solubilized in Cremophor^®^, dissolved in distilled water as needed for each experimental protocol. The final concentration of Cremophor^®^ did not show any interference in the *in vivo* experiments, according to data from previous experiments.

### Participation of K^+^ Channel on the Spasmolytic Activity of LM-EtOH_AP_ on Guinea Pig Ileum

Guinea pigs (n = 5) were fasted for 18 h and euthanized by cervical dislocation followed by the sectioning of the cervical vessels and the ileum was removed, cleaned of adhering fat and connective tissues. Segments of approximately 2 to 3 cm in length were suspended in organ bath (5 ml) and stabilized for 30 min in modified Krebs solution (mM): NaCl (117.0), KCl (4.7), MgSO_4_ (1.3), NaH_2_PO_4_ (1.2), CaCl_2_ (2.5), NaHCO_3_ (25.0), and glucose (11.0) at 37°C and bubbled with a carbogen mixture (95% O_2_ and 5% CO_2_) in a resting tension of 1 g (Daniel et al., 2001).

After stabilization, a contraction was evoked with histamine (10^-6^ M) in both absence (control) and presence of CsCl (5 mM), a non-selective K^+^ channels blocker (Cecchi et al., 1987); glibenclamide (10^-5^ M), a selective ATP sensitive K^+^ channels blocker (K_ATP_) (Sun and Benishin, 1994); 4–aminopyridine (0.3 mM), a selective voltage activated K^+^ channels blocker (K_V_) (Robertson and Nelson, 1994); apamin (100 nM), a selective small–conductance K^+^ channels blocker (SK_Ca_) (Ishii et al., 1997); and TEA^+^ (1 mM), a selective big–conductance K^+^ channels blocker (BK_Ca_) (Knot et al., 1996) in independent experiments, which were added to the organ baths 20 min before the histamine–induced contraction. During the sustained phase of the contraction, LM-EtOH_AP_ (0.1–729 μg/ml) was cumulatively added to obtain a relaxation curve.

The relaxation induced by LM-EtOH_AP_ was expressed as the reverse percentage of the initial contraction induced with the agonist. LM-EtOH_AP_ relaxant potency was measured by the molar concentration of a substance that produces half of its maximum effect (EC_50_) and the maximal relaxant response by the maximum effect (E_max_).

### Toxicological Evaluation

#### Investigation of the Hemolytic Effect of LM-EtOH_AP_ in Rat Erythrocytes

After 12 h of fasting period, a blood sample of rats (250, 500, and 750 µg/ml, n = 3) was collected *via* cardiac puncture, mixed with NaCl 0.9% and CaCl_2_ 10 mM and centrifuged at 2,500 rpm for 5 min (twice) to obtain the erythrocytes. Triton X-100^®^ 1% (100 µl, positive control) or LM–EtOH_AP_ (different concentrations) was added to erythrocytes suspension. The negative control was an erythrocytes suspension plus NaCl 0.9% and CaCl_2_ 10 mM. Hemolysis was quantitated by spectrophotometry at 540 nm and expressed as percentage (Rangel et al., 1997).

#### Evaluation of the Acute Toxicity

After 12 h of fasting period, male (n = 6) and female (n = 6) mice were treated with NaCl 0.9% (10 ml/kg) plus Cremophor^®^ (negative control) or LM-EtOH_AP_ (2,500 or 5,000 mg/kg) orally (p.o.) or (1,000 or 2,000 mg/kg) intraperitoneally (i.p.). General signs and symptoms of toxicity, such as contortions, aggression, sedation, and others were recorded by 4 h. The assessment of these types of behaviors was based on Almeida et al. (1999). The animals were also evaluated up to 24 h and 14 days in order to monitoring lethality and determine the lethal dose of extract to 50% of treated animals (LD_50_). Based on this test, doses for pharmacological studies were determined (Almeida et al., 1999).

### Evaluation of Antidiarrheal Activity of LM-EtOH_AP_ in Mice

#### Effect of LM-EtOH_AP_ on Castor Oil-Induced Diarrhea

After 12 h of fasting period, male and female mice were divided into three groups (n = 6, each) and were treated orally with NaCl 0.9% plus Cremophor^®^ (10 ml/kg, negative control), loperamide (10 mg/kg, positive control), or LM-EtOH_AP_ (125, 250, 500, and 750 mg/kg p.o.). After 30 min of treatment, castor oil was administrated orally (0.01 ml/g) to each animal to induce diarrhea. The animals were separated and placed in individual boxes lined with white paper. Then, the animals were inspected about the number of stools and its consistency for 4 h period, classifying them into solid or liquid and then determined the total number of stools and the number of liquid episodes (Awouters et al., 1978).

The inhibitory effect exerted by LM-EtOH_AP_ was evaluated based on the dose of a drug that produces half of its maximal effect (ED_50_).

#### Effect of LM-EtOH_AP_ on Normal and Castor Oil-Induced Intestinal Transit

Male and female mice were divided into three groups (n = 6, each) and after 12 h of fasting period, were treated orally with NaCl 0.9% plus Cremophor^®^ (10 ml/kg, negative control), atropine (2 mg/kg, positive control), or LM-EtOH_AP_ (125, 250, and 500 mg/kg p.o.). Thirty min later, activated charcoal 5% solubilized in carboxymethylcellulose 0.5% (0.01 ml/g) was administered. The animals were euthanized by cervical dislocation 30 min after administration of activated charcoal, the abdominal cavity opened and the small intestine removed. The total length of the small intestine (distance from the pylorus to the ileocecal valve) and the distance traveled by the activated charcoal were measured and compared (Rao et al., 1997). The results were expressed as a percentage of distance traveled by the marker in relation to total length of small intestine.

In other protocols, the same procedures were made, except that castor oil (0.01 ml/g) was administrated orally 30 min before the activated charcoal (Hsu, 1982; Aye-Than et al., 1989).

#### Effect of LM-EtOH_AP_ Extract on Castor Oil-Induced Intestinal Fluid Accumulation

After 24 h of fasting period, male and female mice (n = 6) were treated orally with NaCl 0.9% (10 ml/kg) plus Cremophor^®^ (negative control), loperamide (10 mg/kg, positive control), or LM–EtOH_AP_ (31.5, 62.5, 125, and 250 µg/ml p.o.). Afterwards, castor oil (2 ml/per animal, p.o.) was administrated and 30 min later the animals were euthanized, the small intestine was dissected, the pylorus to the cecum, the contents expelled, and the volume of the fluid measured (Robert et al., 1976; Di Carlo et al., 1993).

### Statistical Analysis

All results were expressed as percentage of the mean ± standard error of the mean (S.E.M.) and statistically analyzed used the Student’s *t*-test or one-way ANOVA followed by Bonferroni’s post–test, as appropriate, values where significantly different when *p* < 0.05. The ED_50_ and EC_50_ values were calculated by non-linear regression for all experiments (Neubig et al., 2003). All data were analyzed using GraphPad Prism^®^ software version 5.01 (GraphPad Software Inc., San Diego, CA, USA).

## Results

### Chemical Analysis of LM-EtOH_AP_

**Figure 1:**
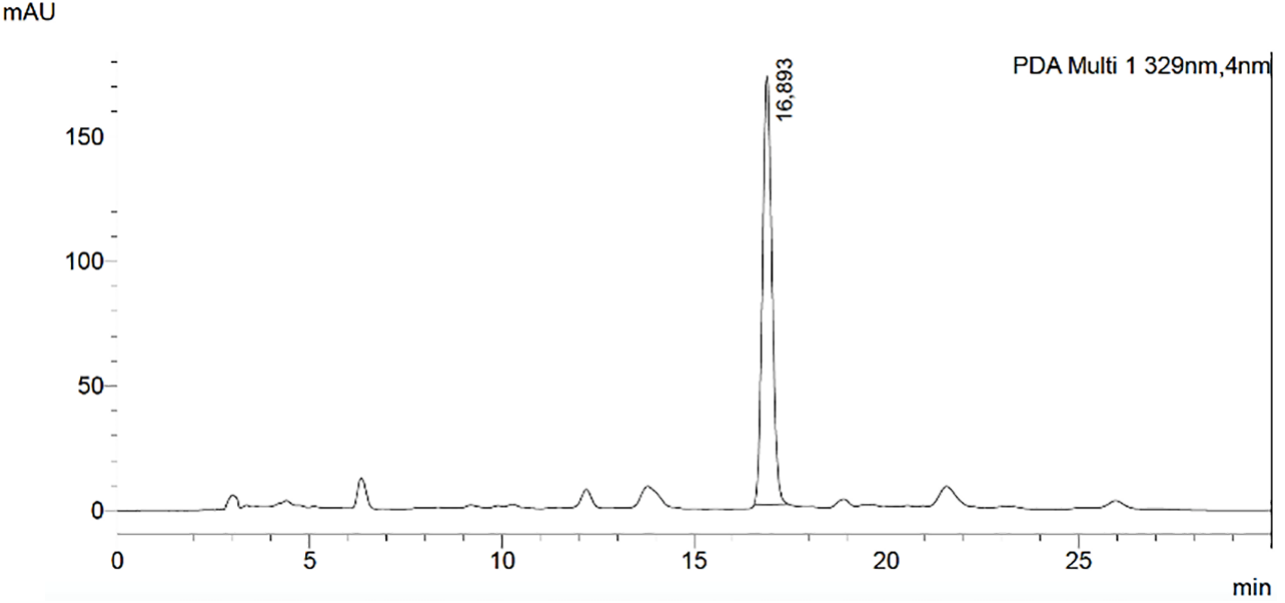
HPLC Chromatogram of the crude ethanolic extract of *Leptohyptis macrostachys*. Chromatographic conditions: Kromasil^®^ 100-C-18 column (250 × 4.6 mm - 5 μm), methanol/water as mobile phase with flow rate of 1 ml/min.

Rosmarinic acid was quantified in the crude ethanolic extract of *Leptohyptis macrostachys* by high-performance liquid chromatographic-diode array detection. Rosmarinic acid (5.44%) was the most abundant phenolic compound, based on this, could be considered a chemical marker. The value of LOD was 2.31 μg/ml and the value for LOQ was 7.71 μg/ml (**Figure 1**).

### Participation of K^+^ Channel on the Spasmolytic Activity of LM-EtOH_AP_ on Guinea Pig Ileum

In the relaxation evaluation, LM-EtOH_AP_ relaxant effect (EC_50_ = 38.9 ± 5.5 μg/ml, n = 5) was attenuated in the presence of 5 mM CsCl (EC_50_ = 82.1 ± 2.1 μg/ml, n = 5). Contrary, LM-EtOH_AP_ relaxant potency was not altered in the presence of 10^–5^ M glibenclamide (EC_50 _= 27.1 ± 2.0 μg/ml, n = 5), 0.3 mM 4–AP (EC_50 _= 30.0 ± 4.3 μg/ml, n = 5), and 100 nM apamin (EC_50 _= 23.9 ± 4.5 μg/ml, n = 5). However, the extract relaxant potency was attenuated in the presence of 1 mM TEA^+^ (EC_50 _= 60.7 ± 3.0 μg/ml, n = 5) (**Table 1**).

**Table 1:** E_max_ (%) and EC_50_ (μg/ml) values of LM-EtOH_AP_ in both the absence and presence of K^+^ channel blockers on guinea pig ileum.

Compounds	E_max_ (%)	EC_50_ (μg/ml)
LM-EtOH_AP_	100.0 ± 0.0	38.9 ± 5.5
5 mM CsCl + LM-EtOH_AP_	100.0 ± 0.0	82.1 ± 2.1^***^
1 mM TEA^+^ + LM-EtOH_AP_	100.0 ± 0.0	60.7 ± 3.0^**^
10^-5^ M glibenclamide + LM-EtOH_AP_	100.0 ± 0.0	27.1 ± 2.0
0,3 mM 4-AP + LM-EtOH_AP_	100.0 ± 0.0	30.0 ± 4.3
100 nM apamin + LM-EtOH_AP_	100.0 ± 0.0	23.9 ± 4.5

Data are expressed as the mean ± S.E.M., respectively (n = 5). Student’s t-test. ^**^p < 0.01; ^***^p < 0.001 (LM-EtOH_AP_ vs. blockers + LM–EtOH_AP_).

### Toxicological Evaluation

#### Investigation of the Hemolytic Effect of LM-EtOH_AP_ in Rat Erythrocytes

**Figure 2:**
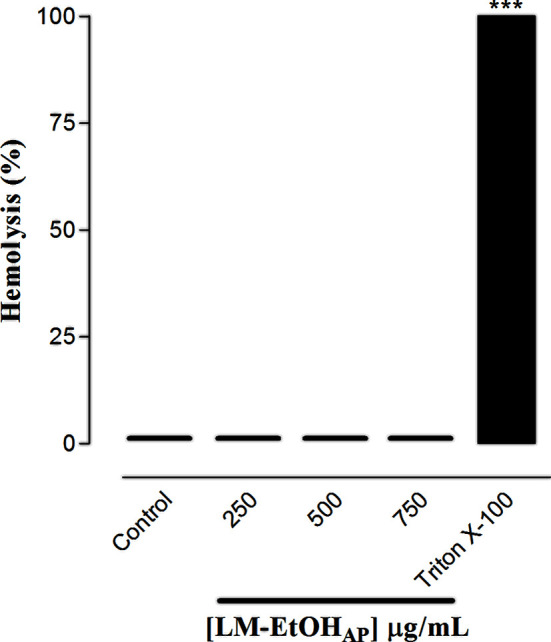
Hemolytic effect of LM-EtOH_AP_ and triton X-100 (positive control) in rat erythrocytes. Columns and vertical bars represent the mean and S.E.M., respectively (n = 3). One-way ANOVA followed by Bonferroni’s post-test, ***p < 0.01 (control vs. triton X-100).

LM-EtOH_AP_ (250, 500, and 750 µg/ml, n = 3) did not induce rat erythrocytes lysis, showing no damage to rat erythrocytes membranes at a range of concentrations used (**Figure 2**).

#### Evaluation of the Acute Toxicity

LM-EtOH_AP_ (2,500 or 5,000 mg/kg p.o. and 1,000 or 2,000 mg/kg i.p.) did not promote behavioral changes in both male and female mice (n = 6, each) throughout the observation period of 4 h. In addition, the extract also did not induce death in the treated animals during the observation period of 14 days.

### Evaluation of Antidiarrheal Activity of LM-EtOH_AP_ in Mice

#### Effect of LM-EtOH_AP_ on Castor Oil-Induced Diarrhea

**Figure 3:**
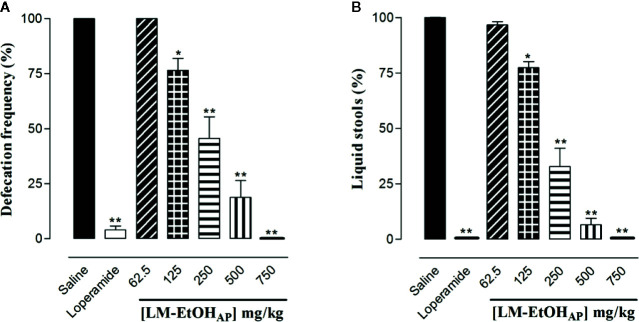
Antidiarrheal effect of LM-EtOH_AP_ on castor oil-induced diarrhea in mice. Percentage of total stool numbers **(A)** and percentage of liquid stools **(B)**. Columns and vertical bars represent the mean and S.E.M., respectively (n = 6). One-way ANOVA followed by Bonferroni’s post–test, ^*^ *p* < 0.05; ^**^ *p* < 0.01 (saline *vs.* loperamide/LM-EtOH_AP_).

LM-EtOH_AP_ (125, 250, 500, and 750 mg/kg p.o., n = 6) inhibited equipotently and in a dose–dependent manner both defecation frequency (ED_50_ = 248.0 ± 41.0 mg/kg) and number of liquid stools (ED_50_ = 201.8 ± 21.7 mg/kg). The E_max_ was achieved at 750 mg/kg, in both parameters, similar to the standard drug loperamide (10 mg/kg) (**Figure 3**).

#### Effect of LM-EtOH_AP_ on Normal and Castor Oil-Induced Intestinal Transit

**Figure 4:**
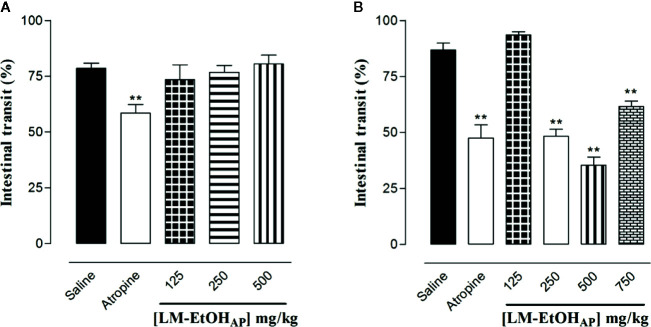
Effect of LM-EtOH_AP_ on the normal **(A)** and castor oil-induced **(B)** intestinal transit in mice. Columns and vertical bars represent the mean and S.E.M., respectively (n = 6). One-way ANOVA followed by Bonferroni’s post-test, ^**^ *p* < 0.01 (saline *vs.* atropine/LM-EtOH_AP_).

LM-EtOH_AP_ (125, 250, and 500 mg/kg p.o., n = 6) did not inhibit the normal intestinal transit. In contrast, atropine (2 mg/kg, p.o., n = 6) decreased the normal intestinal transit from 78.6 ± 2.2% (control) to 58.5 ± 3.8% (**Figure 4A**).

Moreover, LM-EtOH_AP_ (250, 500, and 750 mg/kg p.o., n = 6) inhibited in a dose-dependent manner the castor oil-induced intestinal propulsion (E_max_ = 35.4 ± 3.6%, ED_50_ = 315.3 ± 27.6 mg/kg). In addition, atropine (2 mg/kg p.o., n = 6) decreased the distance traveled by the marker from 86.8 ± 3.1% (control) to 47.4 ± 5.9% (**Figure 4B**).

#### Effect of LM-EtOH_AP_ Extract on Castor Oil-Induced Intestinal Fluid Accumulation

**Figure 5:**
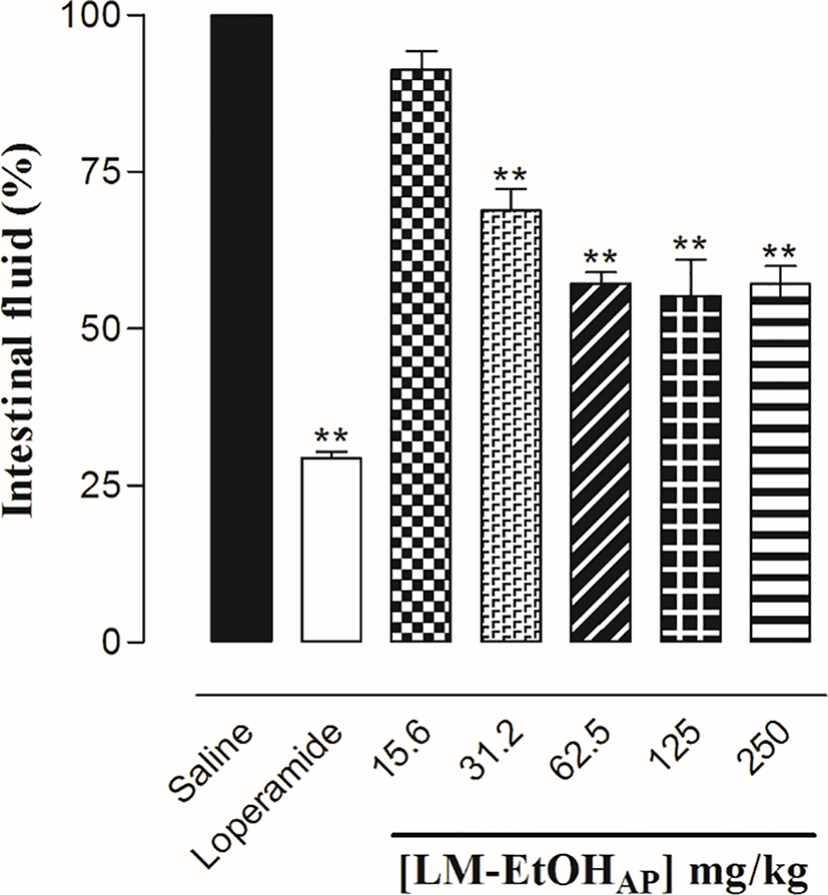
Effect of LM-EtOH_AP_ on castor oil-induced intestinal fluid accumulation in mice. Columns and vertical bars represent the mean and S.E.M., respectively (n = 6). One-way ANOVA followed by Bonferroni’s post-test, ^**^ *p* < 0.01 (saline *vs.* loperamide/LM-EtOH_AP_).

LM-EtOH_AP_ (31.5, 62.5, 125, and 250 µg/ml p.o., n = 6) inhibited in a dose-dependent manner the liquid content (E_max_ = 44.8 ± 5.8%, ED_50_ = 259.9 ± 65.3 mg/kg). Furthermore, loperamide (10 mg/kg p.o., n = 6) decreased the liquid content from 100.0 ± 0% (control) to 29.4 ± 1.0% (**Figure 5**).

## Discussion

In the investigation of this study, we evaluate the role of K^+^ channels in the spasmolytic action mechanism, toxicological and antidiarrheal activities of LM–EtOH_AP_, an extract that contains rosmarinic acid as a chemical marker (**Figure 1**). Moreover, the mechanism underlying its spasmolytic action includes the positive modulation of BK_Ca_ leading to smooth muscle relaxation that it is possibly related to its antidiarrheal activity.

Recently, we reported that LM–EtOH_AP_ has selective spasmolytic action on guinea pig ileum, probably, due to the inhibition of Ca^2+^ influx through voltage-gated calcium channels (Ca_V_) (Souza et al., 2013). Since the contraction of smooth muscle depends on the balance between the increase and decrease of the K^+^ channels activity, leading to membrane hyperpolarization/repolarization or depolarization, respectively, these channels have been shown to regulate the Ca_V_ opening (Thorneloe and Nelson, 2005).

Thereby, we hypothesized that this extract might be inhibiting the Ca^2+^ influx indirectly *via* positive modulation of K^+^ channels. In order to test this hypothesis, the CsCl was used as a pharmacological tool to block K^+^ channels in a non–selective manner (Cecchi et al., 1987). The relaxant potency of LM–EtOH_AP_ was reduced in the presence of this blocker (**Table 1**), indicating the role of K^+^ channels on LM–EtOH_AP_ spasmolytic action. In addition, as the intestinal smooth muscle expresses different subtypes of K^+^ channels, such as BK_ca_, K_ATP_, K_v_, SK_ca_, among others (Vogalis, 2000), we decided to investigate which specific channel was involved in this effect using their selective blockers. The relaxant potency of the extract was reduced in the presence of 1 mM TEA^+^, but not in the presence of glibenclamide, 4–AP and apamin, suggesting that the activation of BK_ca_ promotes relaxation on guinea pig ileum (**Table 1**).

Alteration of intestinal contractility is a process that characterize intestinal colic, constipation, and diarrhea. Clinically, the pain caused by intestinal spasms are often treated with drugs that induce relaxation of smooth muscles (Sato et al., 2007). In folk medicine, the use of species from *Hyptis* genus on intestinal disorders treatment is an old practice reported for *H. suaveolens*, *H. martiusii*, *H. umbrosa*, and *H. verticillata* (Mukherjee et al., 1984; Rojas et al., 1992; Agra et al., 2008). Heretofore, we have shown *in vitro* that LM–EtOH_AP_ presents spasmolytic activity on intestinal smooth muscle, however no *in vivo* evidence of an antidiarrheal activity of this extract has been investigated.

Considering that some studies have reported toxicological effects of *Hyptis* species, such as *H. fruticosa* (Silva et al., 2006), *H. mutabilis* (Forgacs et al., 1983), *H. martiusii* (Caldas et al., 2013), and *H. verticillata* (Picking et al., 2013), we decide to obtain information regarding the cytotoxic profile and possible acute toxicity of LM–EtOH_AP_ using *in vitro* and *in vivo* approach, respectively.

The mechanical stability of the erythrocyte membrane is a good parameter used in cytotoxicity screening, since its structural dynamics favors interactions with drugs that can promote lysis (Sharma and Sharma, 2001). In this study, LM–EtOH_AP_ did not produce damage to rat erythrocyte membranes in the range of concentrations used (**Figure 2**), suggesting that the extract may not presents toxic effects in other stronger cell types.

Similarly, during the observation period of 4 h neither oral dose nor intraperitoneal dose administration of LM–EtOH_AP_ induce behavioral changes, described as characteristics of toxicity and including parameters of hyperactivity, aggressiveness, sedation, convulsions, among others (Almeida et al., 1999). Therefore, these data demonstrate that the extract did not presents toxic signs on the central nervous system. Moreover, there were no deaths of treated animals during the observation period of 24 h and 14 days, making it impossible to determine the LD_50_. Thus, these results give us a safety margin to use LM–EtOH_AP_ in the investigation of a possible *in vivo* antidiarrheal activity.

Currently, international organizations support studies with traditional medicinal practices that seek treatment or prevention of diarrhea (Ventura-Martínez et al., 2011), because this disease is the second leading cause of death in children younger than 5 years old, constituting a public health problem that results in 760,000 children deaths every year (Shafi et al., 2014; WHO, 2019).

Diarrhea is a pathologic condition where the intestinal fluid secretion it is not balanced by absorption, resulting in symptoms as increase of water content, volume, and frequency of stool. In animal models, diarrhea is widely induced using castor oil in order to discover new drugs with potential antidiarrheal activity (Borrelli et al., 2006).

In this study, LM–EtOH_AP_ inhibited castor oil-induced diarrhea regarding both defecation frequency and number of liquid stools (**Figure 3**), suggesting that the extract has active constituents with antidiarrheal activity. These substances may change the bowel motility inhibiting the intestinal transit or increasing water and electrolyte absorption in the gastrointestinal tract (Field and Semrad, 1993). Hence, we investigated if changes in the intestinal transit and/or fluid accumulation are related to the antidiarrheal activity of LM–EtOH_AP_.

Interestingly, the extract did not inhibit the normal intestinal transit, however, atropine, used as positive control, decrease the distance traveled by the activated charcoal (**Figure 4A**). Despite this, LM–EtOH_AP_ inhibited in a dose-dependent manner the castor oil-induced intestinal transit and atropine also decrease the distance traveled by the marker (**Figure 4B**), suggesting that the antidiarrheal effect of the extract involves alterations in the intestinal motility only in pathologic condition.

In diarrhea, there is a pronounced stimulation of cells secretion that becomes greater than the amount capable of being resorbed (Menezes et al., 1994). In castor oil-induced intestinal fluid accumulation model is developed an electrolyte hypersecretory response (Mascolo et al., 1993). Thus, using this protocol we showed that the extract inhibited, in a dose-dependent manner, the intestinal fluid accumulation (**Figure 5**), suggesting that LM–EtOH_AP_ antidiarrheal effect also involves decrease in intestinal secretion. This action is desirable since the main common manifestation of different types of diarrhea is the dehydration and, in many cases, intestinal transit inhibition is not desired because it can delay or prevent the elimination of potential pathogens (Gurgel et al., 2001).

In conclusion, the spasmolytic action of LM–EtOH_AP_, on guinea pig ileum involves the positive modulation of BK_ca_. In addition, the extract does not have cytotoxic effect or systemic toxicity in mice. Interestingly, it is now established that LM–EtOH_AP_ has an antidiarrheal effect, as it inhibits both intestinal motility and secretion. Complementary studies are necessary to better elucidate the action mechanism of this potential antidiarrheal agent. Additionally, it is relevant to conduct studies with rosmarinic acid to verify whether the observed effects are due to its spasmolytic and antidiarrheal potential.

## Data Availability Statement

The raw data supporting the conclusions of this article will be made available by the authors, without undue reservation, to any qualified researcher.

## Ethics Statement

The animal study was reviewed and approved by Ethics Committee in Research (CEP) of UFAL, certificate no. 006775/2011-09, and the Ethics Committee on Animal Use (CEPA/UFPB), certificate no. 0506/05.

## Author Contributions

IS is the author who mainly contributed to this research, performing literature search, pharmacological experiments, analysis of the data, and writing the manuscript. MV, AS, and PS were involved in acquisition, interpretation, and analysis of *in vivo* pharmacological experiments. CV, DA, VC, JT, and MS performed the phytochemical experiments. HP was involved in toxicological experiments. BS and FC were involved in design, interpretation of the data, and review of the manuscript. All authors contributed to the article and approved the submitted version.

## Conflict of Interest

The authors declare that the research was conducted in the absence of any commercial or financial relationships that could be construed as a potential conflict of interest.

## Abbreviations

HPLC–DAD, high-performance liquid chromatographic-diode array detection; LM-EtOH_AP_, crude ethanolic extract obtained from the aerial parts of *Leptohyptis macrostachys*; BK_Ca_, selective big–conductance K^+^ channels blocker; PPgPNSB, Programa de Pós–graduação em Produtos Naturais e Sintéticos Bioativos; CCS, Centro de Ciências da Saúde; UFPB, Universidade Federal da Paraíba; TEA^+^, tetraethylammonium chloride; 4–AP, 4–aminopyridine; CsCl, cesium chloride; K_ATP,_ selective ATP sensitive K^+^ channels blocker; K_V,_ selective voltage activated K^+^ channels blocker; SK_Ca,_ selective small–conductance K^+^ channels blocker; BK_Ca,_ selective big–conductance K^+^ channels blocker; EC_50,_ molar concentration of a substance that produces half of its maximum effect; E_max,_ maximum effect; LD_50,_ the lethal dose of extract to 50% of treated animals; ED_50,_ dose of a drug that produces half of its maximal effect; S.E.M., mean ± standard error of the mean.
